# Chemical Composition and Ixodicidal Activity of *Copaifera reticulata* Ducke Oleoresin and Its Sesquiterpenic (Volatile) and Diterpenic (Resin) Fractions against *Rhipicephalus microplus* Larvae

**DOI:** 10.1021/acsomega.6c00834

**Published:** 2026-06-18

**Authors:** Selino Monteiro Costa Filho, Ana Beatriz Barbosa de Sousa, Poliana Leão Peleja, Gleisson Willen Cerdeira Lemos, Adilson Sartoratto, Elaine Cristina Pacheco de Oliveira, Antonio Humberto Hamad Minervino

**Affiliations:** † Programa de Pós-Graduação em Biociências, Universidade Federal do Oeste do Pará, UFOPA, Santarém 68040-255, PA, Brazil; ‡ Laboratório de Biotecnologia de Plantas Medicinais, LBPM, Universidade Federal do Oeste do Para, Santarém 68040-255, PA, Brazil; § Laboratório de Sanidade Animal, LARSANA, Universidade Federal do Oeste do Pará, Santarém 68040-255, PA, Brazil; ∥ Centro de Pesquisas Químicas Biológicas e Agrícolas, Universidade Estadual de Campinas, Campinas 13148-218, SP, Brazil

## Abstract

The species *Copaifera reticulata* Ducke, native to the Amazon, produces oleoresin rich in terpenoid compounds with potential for controlling *Rhipicephalus microplus*, a cattle tick responsible for significant losses in livestock production. This study aimed to characterize the chemical constituents of the oleoresin (OR) and its sesquiterpenic (volatile, VF) and diterpenic (resin, RF) fractions by GC–MS and to evaluate their ixodicidal activity against *R. microplus* larvae through the Larval Immersion Test (LIT). The oleoresin and volatile fraction were dominated by sesquiterpenes, whereas the resin fraction was enriched in diterpenes. Ixodicidal tests showed that the RF was the most effective fraction, achieving 100% larval mortality at moderate concentrations (LC_50_ = 3.35 mg/mL; LC_95_ = 7.22 mg/mL), followed by the OR (LC_50_ = 8.84 mg/mL; LC_95_ = 27.34 mg/mL), while the VF showed lower activity (LC_50_ = 45.28 mg/mL; LC_95_ = 89.41 mg/mL). These results demonstrate that ixodicidal activity depends on the composition and stability of terpenoids, particularly diterpenes, highlighting the RF as the most effective fraction and reinforcing the potential of *C. reticulata* as a natural alternative for tick control.

## Graphical abstract

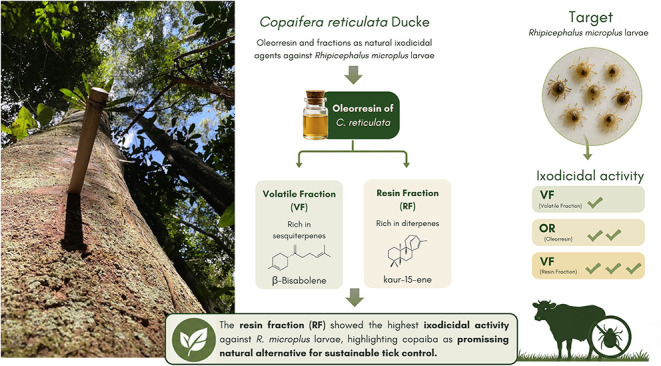

## 1 Introduction

Medicinal plants have historically played an essential role in traditional medicine and remain an important source of bioactive compounds for pharmacological and agricultural applications, and Brazil is globally recognized for its vast biodiversity, considered the country with the greatest biological diversity on the planet.^1^ This biodiversity is accompanied by rich ancestral knowledge, preserved by Indigenous, quilombola, riverside, and family farming communities.^2^ Interest in these ancestral practices has been growing, contributing to their revival and appreciation, which underscores the therapeutic potential of medicinal plants.^3^ Ethnobotanical knowledge has long supported the relevance of plant secondary metabolites as a continuous source of bioactive compounds for modern pharmacological and agricultural applications.^4^

Among the natural products most widely used in Amazonian traditional medicine is copaiba oleoresin. Copaiba trees, belonging to the genus *Copaifera* (Fabaceae, Caesalpinioideae), stand out for their therapeutic properties and economic importance.^5^ Although this genus is widely distributed and well adapted to tropical regions of South and Central America, Africa, and Asia, its greatest biodiversity occurs in Brazil. Approximately 25 species are recorded in the country, of which 16 are endemic, with the highest species richness concentrated in the North and Northeast regions.^6,7^

The species *Copaifera reticulata* Ducke is the most studied within the genus due to its high medicinal potential and proven biological properties.^8^ In addition to being the most abundant species in the Brazilian Amazon,^9^ its wide distribution and the ease of extracting its oleoresin, a natural product of secondary metabolism rich in terpenoid compounds, make it particularly notable. The volatile fraction is composed of sesquiterpenes, such as caryophyllene, while the resin fraction is rich in diterpenes, for example, kaurenoic acid.^10,11^ These compounds are associated with various biological activities due to their wide distribution and therapeutic potential, particularly in antimicrobial, wound-healing, anti-inflammatory, and larvicidal activities.^8,12,13^ The chemical composition of copaiba oleoresin changes substantially according to geographic origin, varying among trees and collection sites, reflecting environmental and edaphic factors.^14^ Thus, the characterization of material from the Tapajós National Forest contributes to understanding regional chemical variability and its biological implications.

Terpenoid classes differ in physicochemical properties that may affect their activity against ticks. Sesquiterpenes are more volatile and may result in transient larval exposure, whereas diterpenes are less volatile and more lipophilic, favoring retention and diffusion through the lipid-rich tick cuticle. Therefore, diterpene-rich resin fractions are expected to produce a more persistent ixodicidal effect than sesquiterpene-rich fractions.^15,16^ Recently, the oleoresin has been investigated for its acaricidal activity against the cattle tick *Rhipicephalus microplus*, a parasite that causes significant losses in livestock due to increasing resistance to conventional acaricides.^17,18^ *R. microplus* is a monoxenous tick of Asian origin widely distributed in tropical and subtropical regions, including Brazil, where it was likely introduced through cattle transportation. Commonly known as the cattle tick (Southern cattle tick; Asian blue tick), this species is a hematophagous arthropod belonging to the family Ixodidae. This parasite is responsible for significant economic losses in livestock worldwide. In Brazil alone, infestations are estimated to cause losses of approximately US$3.24 billion annually through reductions in productivity^19^ and acts as a vector for pathogens such as *Babesia bovis*, *Babesia bigemina*, and *Anaplasma marginale*.^20^

The control of *R. microplus* relies heavily on synthetic acaricides, whose indiscriminate application has promoted resistance and generated significant environmental and toxicological impacts.^21,22^ Compounds such as organophosphates, carbamates, and organochlorines are associated with carcinogenicity and bioaccumulation in aquatic ecosystems.^23^ The increasing resistance of *R. microplus* to multiple commercial acaricides poses a significant challenge, with records reported in several countries worldwide,^24,25^ including in the Lower Amazon,^26^ and in western Pará, where heterogeneous susceptibility profiles to amitraz, ivermectin, and doramectin have recently been described,^27^ increasing the need for alternative control strategies.^28^

The search for safe and sustainable alternatives has driven the use of natural products as strategies for parasite control. In this context, oleoresins from the *Copaifera* genus stand out as promising sources of bioactive compounds with acaricidal potential. The oleoresin of *C. reticulata* presents itself as an innovative alternative for the control of *R. microplus*. However, the relative contribution of sesquiterpene and diterpene fractions of copaiba oleoresin to ixodicidal activity remains unclear, and comparative evaluations of separated fractions are still limited. This approach allows the biological activity to be interpreted in relation to terpene class, providing insight into which chemical fraction is primarily responsible for the ixodicidal effect. Thus, this study aimed to characterize its chemical composition and evaluate the in vitro ixodicidal activity of the oleoresin and its volatile and resin fractions against *R. microplus* larvae.

## 2 Materials and Methods

### 2.1 Plant Material

The oleoresin of *C. reticulata* Ducke was obtained from an adult tree located in the Tapajós National Forest (FLONA do Tapajós), Belterra-Pará Brazil (S 03° 21′ 09.9″ and W 55° 01′ 26.5″), at kilometer 117 of the BR-163 highway. The collection was carried out under authorization from the Biodiversity Authorization and Information System (SISBIO 68739) and the National System for the Management of Genetic Heritage and Associated Traditional Knowledge (SISGen AD2B196). A voucher specimen was collected and deposited at the IAN Herbarium (Herbário do Instituto Agrônomico do Norte), Embrapa Amazônia Oriental (Empresa Brasileira de Pesquisa Agropecuária), Belém, Pará, Brazil, under voucher number IAN201211. The species identification was performed by a qualified botanist, and the voucher is public accessible in the herbarium collection. Only one tree was sampled, and a single collection was performed during one harvesting period. Oleoresin sampling was performed by drilling two holes in the trunk at heights of 1.0 and 1.5 m using a 2 cm diameter auger, following standard copaiba tapping procedures described in the literature.^29^ The samples were stored in amber flasks, protected from light, sealed, labeled, and kept at room temperature. The hydrodistillation procedure was performed based on,^30^ with minor operational adaptations to accommodate laboratory. The main adaptations included the use of a 3 L round-bottom flask, adjustment of the oleoresin-to-water ratio (100 mL of oleoresin to 1.8 L of distilled water), and a distillation time of 6 h. Additionally, the condenser operating conditions (water circulation at 10 °C with a flow rate of 4 L/min) were specified to ensure reproducibility. No other modifications to the original methodology were made. Photographs documenting the plant material, oleoresin extraction, and bioassay procedures are provided in the Supporting Information (Figures S1–S3).

### 2.2 Tick Collection

Tick collection was carried out at a slaughterhouse in the municipality of Santarém, Pará, through the inspection of cattle destined for slaughter, sourced from a farm from Belterra municipality. Engorged females were collected from adult Nelore-type cattle (*Bos indicus*), aged between 24 and 36 months. Ticks were manually collected without interfering with the slaughter process. The entire procedure was conducted with authorization from the slaughterhouse and the Ethics Committee on Animal Use (CEUA-UFOPA), under protocol n° 0920240318, by a trained team following the site’s established safety protocols.

The collected females were transported to the Animal Health Laboratory (LARSANA) at the Federal University of Western Pará (UFOPA), where they were identified using a morphological key and stored until the tests. The ticks were washed in distilled water and dried on paper towels. After drying, ten female ticks were placed in plastic Petri dishes and kept in a BOD incubator at a temperature of 27–28 °C and 80–90% relative humidity for 14 days to allow egg-laying. After oviposition, the eggs from each Petri dish were homogenized, and 250 mg aliquots were placed in 10 mL syringes adapted for the purpose. The syringe ends were sealed with hydrophilic cotton stoppers, allowing air and moisture circulation while preventing larvae escape. The syringes were then stored in a BOD incubator under the same environmental conditions to enable egg hatching and larvae development. The larvae used in the tests were 14 to 21 days old and unfed.

### 2.3 GC-MS Analysis

Prior to injection into the GC–MS system, derivatization was applied only to the oleoresin and the resin fraction in order to enhance the detection of diterpenic constituents, as described by Migowska et al.^31^ Approximately 30 mg of each sample was homogenized in 7 mL of a 10% acetone/methanol solution, with the addition of 200 μL of trimethylsilyl diazomethane (TMSD). The mixture was stirred for 1 h at room temperature, concentrated to dryness in a rotary evaporator, and resuspended in ethyl acetate for subsequent injection into the chromatograph. The samples were analyzed using a GC-MS system with an injector at 250 °C, a split ratio of 1:50, and a flow rate of 1.0 mL/min of ultrapure helium, with the detector set at 300 °C. The oven temperature followed a ramp starting at 110 °C, increasing at 5 °C/min to 300 °C, and held for 20 min. Ionization was performed by electron impact at 70 eV, with a scan range of 40 to 600 amu. Linear Retention Index (RI) were calculated relative to a homologous series of *n*-alkanes (C8–C24) analyzed under identical chromatographic conditions and were determined according to the Van den Dool and Kratz equation.^32^ Constituent identification was conducted by comparing experimental RI values with those reported in the literature and by matching mass spectra with the equipment’s electronic library (NIST-11).^33,34^

### 2.4 Larval Immersion Test (LIT)

The LIT was performed following the procedure established by the Food and Agriculture Organization of the United Nations^35^ with modifications by Sabatini et al.^36^ Three samples of *C. reticulata* Ducke were evaluated: crude oleoresin (OR), volatile fraction (VF), and resin fraction (RF). Due to differences in density among the samples, the final concentrations in mg/mL were specific to each fraction and are presented in Table 1.

**Table 1:** Tested Concentrations Expressed in % (v/v) and in mg/mL for Each Fraction

natural product concentration (%) v/v	OR (mg/mL)	VF (mg/mL)	RF (mg/mL)
0.3125%	2.83	2.83	3.28
0.625%	5.66	5.66	6.56
1.25%	11.33	11.31	13.13
2.50%	22.65	22.63	26.25
5%	45.3	45.25	52.5
10%	90.6	90.5	105

The samples were prepared at their specific concentrations and added to 2.5 mL Eppendorf tubes. Larvae (unfed), originating from standardized egg masses (250 mg), were immersed in the test solutions using a brush and gently vortexed for 5 min. The larvae were then transferred onto filter paper envelopes, sealed with paper clips, and incubated in a BOD chamber at 27 ± 1 °C and relative humidity >80 ± 10% for 24 h. After this period, the number of live and dead larvae was counted with the aid of a vacuum pump to facilitate handling. Following the criteria described elsewhere,^37^ larvae exhibiting leg movements but lacking locomotion capacity were considered dead. The assays were conducted in triplicate, using six increasing concentrations of each Copaiba product (OR, VF, and RF), prepared in 1% Tween 80 (v/v). The positive control consisted of a commercial acaricide formulation (Colosso Pulverização, Ourofino Saúde Animal; cypermethrin 15% + chlorpyrifos 25%) diluted according to the manufacturer’s recommendation (1:800; 0.125% v/v) in 1% Tween 80 solution, while 1% Tween 80 served as the negative control.

### 2.5 Data Analysis

The mortality of larvae was expressed as a percentage, considering the average mortality rates observed for each tested sample at its different concentrations. For the calculation of corrected mortality, the Abbott formula was applied. The mortality observed in the negative control was 5.8%, remaining below the 10% threshold recommended.

$$\mathrm{mortality}\;\left(\%\right)=\frac{\mathrm{deade}\;\mathrm{larvae}\times 100}{\mathrm{total}\;\mathrm{larvae}}$$

$$corrected\;mortality\;\left(\%\right)=\frac{\left(\%\mathrm{mortality}\;\mathrm{of}\;\mathrm{the}\;\mathrm{test}-\%\mathrm{mortality}\;\mathrm{of}\;\mathrm{the}\;\mathrm{control}\right)}{\left(100-\%\mathrm{mortality}\;\mathrm{of}\;\mathrm{the}\;\mathrm{control}\right)}\times 100$$

The Probit analysis was conducted with the bioassay results data using MedCalc version 23.2.1 (MedCalc Software Ltd., Ostend, Belgium). Lethal concentrations with 95% confidence intervals (CIs) were estimated.

## 3 Results

### 3.1 Chemical Characterization of the Oleoresin and Its Fractions by GC-MS

The oleoresin, as well as its fractions, were analyzed by GC-MS, and the corresponding total ion chromatograms are shown in Figure 1. The chemical constituents were identified based on their mass spectra and compared with robust literature data and an electronic library, in addition to retention indices. The detailed composition of the identified compounds is presented in Table 2.

**Figure 1:**
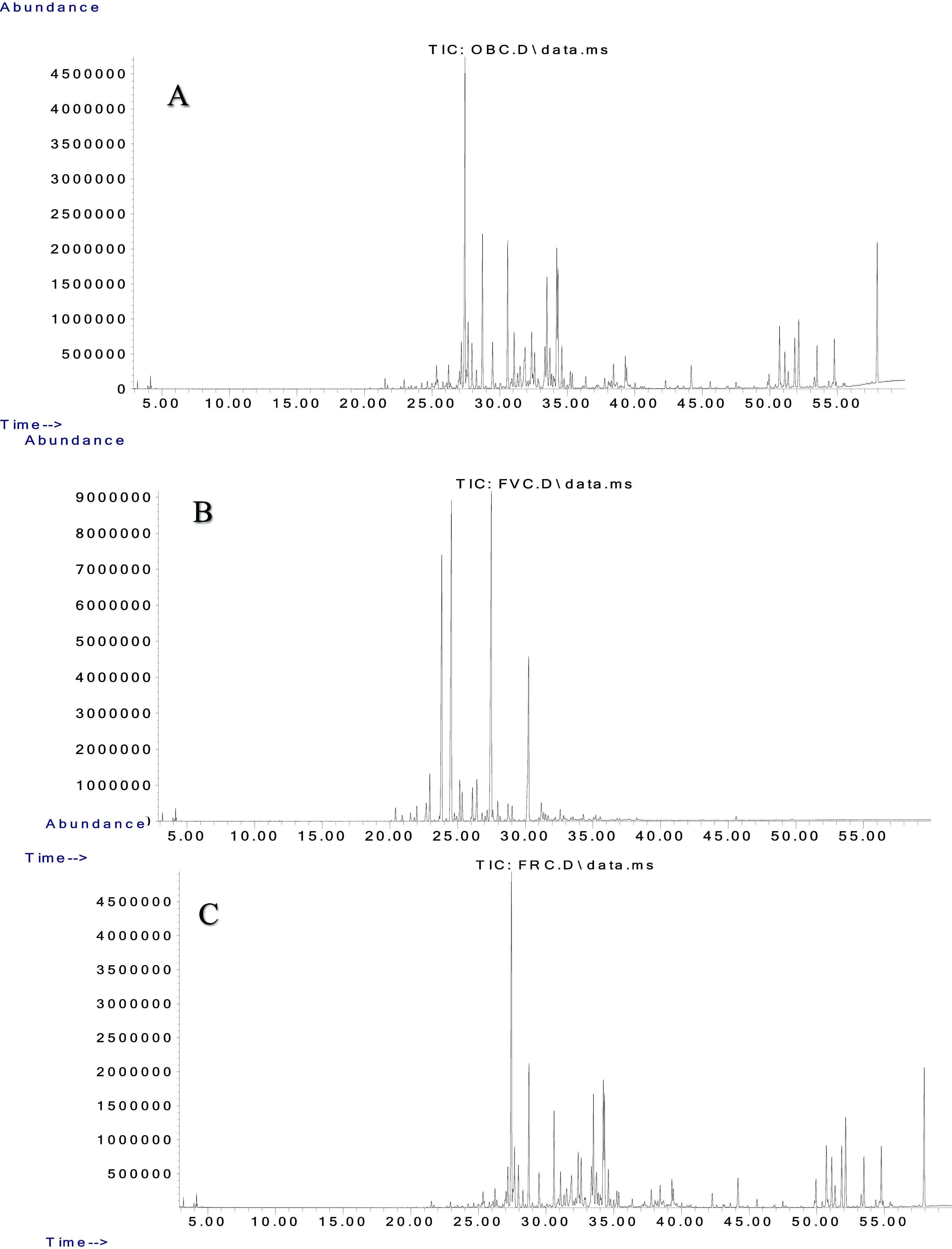
Total ion chromatograms of (A) oleoresin, (B) volatile fraction, (C) resin fraction from *C. reticulata*.

**Table 2:** Major Compounds Found in the Crude Oleoresin of *C. reticulata* Ducke and Its Volatile and Resin Fractions^a^

constituent	RT (min)	RI	% OR	% VF	% RF
*cis*-α-Bisabolene	27.17	1502	1.78		
β-Bisabolene	27.44	1509	15.64		
*cis*-γ-Bisabolene	27.67	1515	2.93		
δ-Cadinene	27.96	1523	1.80		
*Sesquiterpene hydrocarbon (*M* = 204)	28.73	1543	5.97		5.97
*Oxygenated sesquiterpene (*M* = 236)	33.50	1669	4.94		4.94
*Sesquiterpene hydrocarbon (*M* = 204)	34.23	1689	5.50		5.50
*Sesquiterpene hydrocarbon (*M* = 204)	34.32	1691	4.35		4.35
kaur-15-ene	44.18	1984	1.41		1.41
Oxygenated diterpene (fragmentation pattern) (*M* = 318)	57.95		6.53		6.53
Cyperene	22.94	1397		2.43	
*trans*-Caryophyllene	23.83	1419		17.65	
α-*trans*-Bergamotene	24.54	1437		22.79	
α-Humulene	25.16	1452		2.04	
β-Bisabolene	27.50	1511		26.37	
Caryophyllene oxide	30.25	1582		12.06	

a RT = retention time (min); RI = linear retention index; OR = oleoresin; RF = resin fraction; VF = volatile fraction. The percentages refer to the relative peak areas (%); *Peaks at *m*/*z* 204, 236, and 318 were classified at the class level based on molecular mass and fragmentation data; no specific structures were assigned due to lack of reference standards.

The results of Gas Chromatography coupled with Mass Spectrometry (GC-MS) showed that the major compounds identified in the crude oleoresin (OR) were β-bisabolene (15.64%), *M* = 318 (6.53%), and *M* = 204 (5.97%). Other constituents, such as *cis*-α-bisabolene (1.78%) and δ-cadinene (1.80%), were also identified. Constituents present in smaller proportions, such as *cis*-γ-bisabolene (2.93%) and kaur-15-ene (1.41%), were also detected, contributing to the chemical diversity of the oleoresin. It is noteworthy that some diterpenic constituents could not be identified due to the absence of compatible data in the consulted libraries and literature.

In the volatile fraction, rich in sesquiterpene compounds (C_15_H_24_), the main compounds identified were β-bisabolene (26.37%), α-*trans*-bergamotene (22.79%), *trans*-caryophyllene (17.65%), and caryophyllene oxide (12.06%). Other constituents were found, such as cyperene (2.43%) and α-humulene (2.04%).

The resin fractionwas enriched in diterpenes relative to the volatile fraction. Among the identified constituents, the oxygenated diterpene at M = 318 (6.53%) and kaur-15-ene (1.41%) corresponded to diterpenic compounds. Based on fragmentation patterns and retention behavior, these ions are consistent with diterpene skeletons commonly reported for *Copaifera* oleoresins such as copalic and hardwickiic acids.^38,39^

### 3.2 Ixodicidal Activity

Table 3 presents the mortality results of the samples after 24 h, including corrected mortality, as well as data for the positive and negative controls in *R. microplus* larvae. The crude oleoresin (OR) and the resin fraction (RF) exhibited significant ixodicidal activity against *R. microplus* larvae, with mortality rates exceeding 95% at concentrations ≥11.33 mg/mL for OR and ≥13.13 mg/mL for RF, corresponding to 1.25% (v/v). In contrast, the volatile fraction (VF) did not reach 95% mortality at any of the tested concentrations.

**Table 3:** Mortality Results of Samples After 24 h against *R. microplus* Larvae^a^

sample	concentration (mg/mL)	mortality (%)	corrected mortality (%)
OR	2.83	15.4	10.2
	5.66	51.5	48.5
	11.33	100	100.0
	22.65	97.2	97.0
	45.30	99.7	99.8
	90.60	98.6	98.5
VF	2.83	11.88	6.5
	5.66	3.86	<0
	11.31	5.25	<0
	22.63	16.6	11.6
	45.25	69.0	67.1
	90.5	87.7	86.9
RF	3.28	66.3	64.2
	6.56	83.2	82.2
	13.13	100	100.0
	26.25	100	100.0
	52.5	99.8	99.8
	105.0	100	100.0
Negative Control		5.8	
Positive Control		100	

a Values indicated as “<0” in the corrected mortality indicate that the larval mortality was lower than that of the negative control, reflecting an absence of effect.

The ixodicidal activity of the crude oleoresin (OR), volatile fraction (VF), and resin fraction (RF) of *C. reticulata* Ducke against *R. microplus* larvae was evaluated through Probit analysis, which enabled the estimation of lethal concentrations (LC) and their respective 95% confidence intervals. The results highlight differences in the potential among the samples, as presented in Table 4. The LC50 and LC95 were determined with quality of fit data (*R* ^2^) on a slope with a 95% confidence level.

**Table 4:** LC_50_ and LC_95_ (mg/mL; 95% Confidence Intervals in Parentheses) for the Crude Oleoresin (OR), Volatile Fraction (VF) and Resin Fraction (RF) of *C. reticulata* Ducke against *R. microplus* Larvae^a^

sample	LC_50_ (95% CI)	LC_95_ (95% CI)	*R* ^2^	*p*-value
OR	8.84 (8.02–9.48)	27.34 (26.70–27.99)	0.59	<0.0001
VF	45.28 (32.10–75.60)	89.41 (64.40–166.10)	0.51	<0.0001
RF	3.35 (3.16–3.56)	7.22 (7.03–7.40)	0.76	<0.0001

a LC_50_ = lethal concentration for 50% of the larvae; LC_95_ = lethal concentration for 95% of the larvae; CI = confidence interval (95%). OR = crude oleoresin; VF = volatile fraction; RF = resin fraction.

The resin fraction (RF) demonstrated the highest potency, with an LC_50_ of 3.35 mg/mL and an LC_95_ of 7.22 mg/mL, reinforcing pronounced ixodicidal efficacy even at low concentrations. The crude oleoresin (OR) exhibited intermediate activity, with an LC_50_ of 8.84 mg/mL and an LC_95_ of 27.34 mg/mL. In contrast, the volatile fraction (VF) showed the lowest activity, with an LC_50_ of 45.28 mg/mL and an LC_95_ of 89.41 mg/mL.

The dose–response curves were determined for the crude oleoresin (OR), volatile fraction (VF), and resin fraction (RF) of *C. reticulata* Ducke, aiming to evaluate the relationship between the sample concentrations (mg/mL) and the mortality of *R. microplus* *larvae* (%). Figure 2A–C illustrates the dose–response curves for the tested samples.

**Figure 2:**
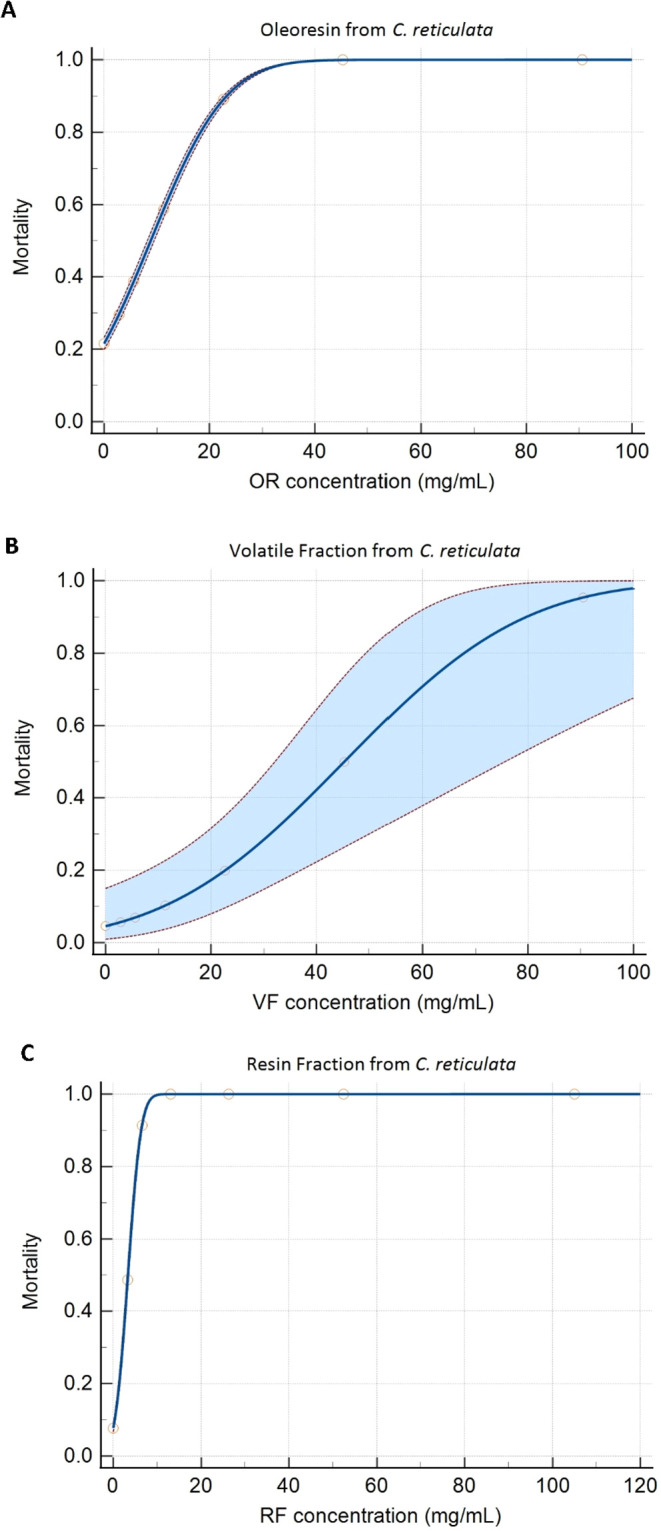
(A) Susceptibility of *R. microplus* Larvae to *C. reticulata* oleoresin after 24 h of Exposure. The circles represent the mortality at each tested concentration, and the solid line corresponds to the Probit regression model. (B) Susceptibility of *R. microplus* Larvae to volatile fraction of *C. reticulata* after 24 h of Exposure. The circles represent the mortality at each tested concentration, and the solid line corresponds to the Probit regression model. (C) Susceptibility of *R. microplus* Larvae to resin fraction of *C. reticulata* After 24 h of Exposure. The circles represent the mortality at each tested concentration, and the solid line corresponds to the Probit regression model.

The proposed mechanism underlying the differential ixodicidal activity among fractions is conceptually illustrated in Figure 3.

**Figure 3:**
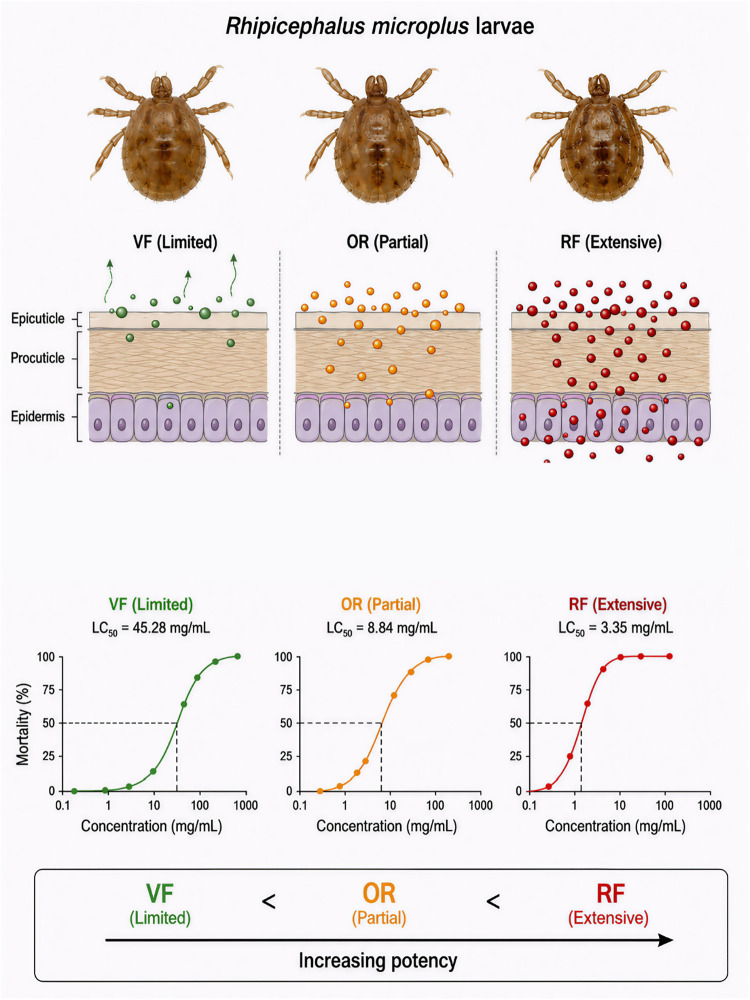
Proposed mechanism of action illustrating the differential cuticular penetration of *C. reticulata* Ducke fractions in *R. microplus* larvae and their corresponding dose–response curves. Upper panel: conceptual cross-section of the larval integument (epicuticle, procuticle, and epidermis) depicting limited penetration for the volatile fraction (VF; green), partial penetration for the crude oleoresin (OR; orange), and extensive penetration for the resin fraction (RF; red), based on the known physicochemical differences between sesquiterpene- and diterpene-rich fractions; this representation is conceptual. Lower panel: dose–response curves (Probit model) for each fraction, with LC_50_ values of 45.28 mg/mL (VF), 8.84 mg/mL (OR), and 3.35 mg/mL (RF), reflecting increasing ixodicidal potency in the order VF < OR < RF.

## 4 Discussion

Native Brazilian plants have been extensively studied for their potential as natural acaricides, capable of controlling parasites of agricultural importance.^40^ Among them, *R. microplus*, a cattle tick responsible for significant losses in livestock production, stands out.^19^ Given the chemical diversity and potential biotechnological applications, the oleoresin of *C. reticulata* Ducke and its fractions emerge as promising alternatives for the management of this ectoparasite.

The chemical characterization of the crude oleoresin (OR) of *C. reticulata* Ducke revealed a composition rich in sesquiterpene compounds, including β-bisabolene, *cis*-γ-bisabolene, *cis*-α-bisabolene, and δ-cadinene; result similar to Barbosa et al.^41^ Moreover, the presence of β-bisabolene in our samples is consistent with the results of Bardají et al.^42^ who identified the same constituent (24.91%) along with trans-α-bergamotene (21.99%) in oleoresin samples. Previous studies indicate that sesquiterpenes constitute a significant portion of the chemical composition of the *Copaifera* genus.^41^ In contrast, diterpenes, although present, are less prevalent.^15^

The main compounds identified in the volatile fraction (VF) were β-bisabolene, α-*trans*-bergamotene, *trans*-caryophyllene, and caryophyllene oxide, in addition to minor constituents such as cyperene and α-humulene. These results corroborate with studies for *C. reticulata* species,^18^ as well as the observations of da Silva et al.,^43^ who reported the presence of α-copaene, β-caryophyllene, α-bergamotene, and β-bisabolene in different species of the genus. Similarly, Xavier-Junior et al.^34^ identified β-bisabolene, β-caryophyllene, and α-bergamotene as the main constituents of *Copaifera* essential oil, reinforcing the chemical profile observed in this study and confirming the consistency of these sesquiterpenes as characteristic markers of the genus. The observed composition is consistent with the chemical profile described for oleoresins of *Copaifera* native to the Amazon, in which sesquiterpenes such as β-bisabolene, α-*trans*-bergamotene, β-caryophyllene, caryophyllene oxide, α-copaene, and α-humulene are frequently reported and considered chemical markers.^44^ Studies conducted in different regions of the Amazon indicate that β-bisabolene and α-*trans*-bergamotene predominate in *C. reticulata* samples from Pará, while β-caryophyllene and β-selinene are more abundant in samples from Amapá.^38,39^

In the resin fraction (RF), the main identified diterpenic compounds were m/z 318 and kaur-15-ene, while a sesquiterpene-class peak (m/z 204) was also detected. Although elevated injector (250 °C) and final oven temperatures (300 °C) may theoretically favor thermal transformation of labile diterpenes, no chromatographic evidence of degradation artifacts (e.g., abnormal peak broadening or formation of secondary low-molecular-weight products) was observed. Moreover, similar temperature programs have been widely applied in GC–MS analyses of Copaifera oleoresins and related diterpenic fractions without reports of thermal instability.^30,45^ Therefore, the analytical conditions were considered suitable for the chemical characterization performed.

The results obtained in this study are consistent with the observations of de Mello et al.^46^ who identified diterpenic acids such as copalic acid, hardwickiic acid, and hydroxycopalic acid in the chemical composition of Copaifera oleoresins. Copalic acid was reported as a common constituent among different species, emphasizing its relevance as a chemical marker for the genus.^30^ Although not all diterpenes in the resin fraction were individually identified in the present study, the observed peaks and corresponding molecular ions strongly suggest the presence of diterpenic structures, reinforcing the chemical complexity and characteristic variability of the genus.^30^

The use of natural products with ixodicidal activity stands out for offering a sustainable and environmentally low-impact approach to pest control, especially in cases of reported resistance to commercial acaricides,^47,48^ including *R. microplus* populations from western Pará recently shown to exhibit resistance to macrocyclic lactones and amitraz. Similar studies have been reported by^17^ and,^49^ who noted high in vitro ixodicidal activity of various medicinal plants. In addition to its traditional use as an antimicrobial,^13,50^ antinociceptive,^51^ and anti-inflammatory agent,^52^ the oleoresin produced by *Copaifera* species also stands out for its potential as a repellent.^53^

The crude oleoresin (OR) exhibited high ixodicidal activity, with mortality starting at a concentration of 11.33 mg/mL and reaching 100% at the highest concentrations. This effect can be attributed to the synergistic action between sesquiterpenes and diterpenes present in the sample, which enhance the biological response.^54^ The resin fraction (RF) stood out as the most active, showing mortality rates above 60% at the lowest concentrations (3.28 mg/mL) and achieving 100% between 13.13 and 105 mg/mL, a result likely associated with the higher concentration of diterpenes, a terpenic class widely recognized for its diverse biological activities,^55^ which justifies the growing scientific interest in these compounds over the past decade.^9^ The comparison among OR, VF, and RF should be interpreted as fraction activity rather than equivalent active-compound dosing. The normalization by mass allows dose expression, but each fraction presents a distinct chemical composition and therefore different effective concentrations of bioactive constituents.^15^ Accordingly, this study represents a preliminary evaluation of complex fractions, not purified molecules, and the observed differences reflect fraction-specific potency rather than direct equipotent exposure. The negative control (Tween 80 at 1%) showed minimal mortality (5.8%), while the positive control achieved 100%, confirming the reliability of the assay. In contrast, the volatile fraction (VF) exhibited significantly lower activity, with near-zero mortality at the lowest concentrations (2.83 to 11.31 mg/mL) and a maximum of 86.9% at 90.5 mg/mL. This lower efficacy may be related to the instability of sesquiterpenes, highly volatile compounds sensitive to external factors such as temperature.^16^ Similar behavior has been reported for essential oils from different *Copaifera* species, in which biological activity is strongly influenced by volatilization, chemical stability, and synergistic interactions among sesquiterpenoid constituents, often resulting in reduced efficacy when these compounds are evaluated as isolated volatile fractions rather than as part of more stable matrices.^56^ Additionally, the absence of diterpenes in this fraction may explain its lower efficiency.^55^

To further characterize the mode of action of the tested fractions, future studies should include morphological observations of treated larvae by optical microscopy or scanning electron microscopy. Such analyses could reveal structural alterations in larval tissues, integument, and cuticle following exposure to the oleoresin and its fractions, providing complementary evidence for the mechanisms underlying the observed ixodicidal activity.^57^

Previous study^17^ reported LC_50_ and LC_99_ values of 1.58 and 3.49 mg/mL, respectively, indicating the acaricidal potential of copaiba oleoresin. In the same study, the authors reported 99% mortality of immature forms of *R. microplus*, including the larval stage. Similarly, Volpato et al.^18^ evaluated the effects of essential oils on different life stages of *R. microplus* and verified that Copaiba essential oil exhibited 71.6% to 86.7% efficacy, reducing hatching and promoting total mortality at both concentrations.

The sesquiterpene methoxy-clovan-9-ol, isolated from *Eugenia copacabanensis*, demonstrated *R. microplus* larval mortality of 93% at a dose of 50 mg/mL, in addition to significant effects on the reproduction of engorged females.^58^ The low LC_50_ values indicate that structurally stable and distinct sesquiterpenes, such as 2-methoxy-clovan-9-ol, exert acaricidal effects comparable to or greater than those of diterpenes, including direct impacts on egg viability. These differences suggest that not only the terpenic class but also the specific chemical nature and stability of the compounds play a determining role in ixodicidal activity.

There is significant variability in the chemical composition of *Copaifera* species, which can be influenced by edaphoclimatic factors, seasonal variations, genetic and epigenetic factors, and biotic interactions with the environment, explaining the difficulty in identifying compounds.^30,59^ Although there is variation in composition and relative abundance of chemical constituents, certain compounds are frequently shared among species of the genus. Consequently, this variation in chemical profiles results in samples with distinct properties and potential applications, as the chemical identity is directly related to the diversity of biological activities observed in the oleoresin samples of *C. reticulata* Ducke and its fractions, including ixodicidal efficacy.

The obtained results reinforce the potential of the oleoresin and, in particular, the resin fraction of *C. reticulata* Ducke as promising candidates for the development of alternative strategies for *R. microplus* control. The pronounced ixodicidal efficacy, associated with low environmental persistence and reduced toxicity to animals, highlights the relevance of these natural products compared to conventional synthetic acaricides.^55^ In this regard, further studies could enhance the understanding of the mechanisms of action involved and evaluate the feasibility of formulations applicable to livestock production systems.

## 5 Conclusion

The obtained results demonstrated that the oleoresin and its VF and RF fractions exhibited a chemical composition consistent with findings in the literature. Both the crude oleoresin (OR) and the resin fraction (RF) of *C. reticulata* Ducke showed pronounced ixodicidal efficacy against *R. microplus* larvae. The difference in performance is related to the composition and stability of the terpenic compounds present, particularly the diterpenes. These results reinforce the potential of natural products as sustainable alternatives for tick management. Thus, *C. reticulata* emerges as a promising option for the control of *R. microplus*.

## Supplementary Material



## Data Availability

The data sets supporting the results of this study are available upon request from the corresponding author.

## Notes

Copaíba (Copaifera reticulata Ducke), (A) tree individual in its natural environment, highlighting the typical tall stature of the species, (B) trunk perforation for oleoresin extraction, demonstrating the traditional tapping method, (C) branch with compound leaves used for botanical identification, (D) freshly collected copaíba oleoresin, showing its color and physical appearance (Figure S1); processing and evaluation of copaíba oleoresin (Copaifera reticulata Ducke), (A) hydrodistillation setup used to separate the resinous fraction from the volatile fraction, (B) resinous fraction obtained after distillation, showing phase separation, (C) preparation and dilution of samples for experimental assays, (D) larval bioassays performed to evaluate the biological activity of the samples (Figure S2); acaricidal bioassay with copaíba oleoresin fractions (Copaifera reticulata Ducke), (A) filter papers containing larvae after immersion in treatments, being transferred to a BOD incubator, (B) experimental setup inside the incubator under controlled conditions, (C) treatment with the resinous fraction, showing dead and desiccated Rhipicephalus microplus larvae, (D) control treatment with Tween (diluent), where larvae remain alive and active (Figure S3) (PDF)
