# Supplementary material for: Chemical Composition and Ixodicidal Activity of Copaifera reticulata Ducke Oleoresin and Its Sesquiterpenic (Volatile) and Diterpenic (Resin) Fractions against Rhipicephalus microplus Larvae
Source: ACS Omega. 2026 Jun 18;11(26):38625–35. doi: 10.1021/acsomega.6c00834 (PMC13347660; doi:10.1021/acsomega.6c00834)

Supporting Information

Chemical composition and Ixodicidal Activity of *Copaifera reticulata* Ducke Oleoresin and Its Sesquiterpenic (Volatile) and Diterpenic (Resin) Fractions Against *Rhipicephalus microplus* Larvae

Selino Monteiro Costa Filho[1,2]; Ana Beatriz Barbosa de Sousa[1,3]; Poliana Leão Peleja[1,3]; Gleisson Willen Cerdeira Lemos[2]; Adilson Sartoratto[4]; Elaine Cristina Pacheco de Oliveira[2]; Antonio Humberto Hamad Minervino[3]

1. Programa de Pós-Graduação em Biociências, Universidade Federal do Oeste do Pará, UFOPA, Santarém 68040-255, PA, Brazil
2. Laboratório de Biotecnologia de Plantas Medicinais, LBPM, Universidade Federal do Oeste do Para, Santarém, 68040-255, PA, Brazil
3. Laboratório de Sanidade Animal, LARSANA, Universidade Federal do Oeste do Pará, Santarém, 68040-255, PA, Brazil
4. Centro de Pesquisas Químicas Biológicas e Agrícolas, Universidade Estadual de Campinas, Campinas, 13086-002, SP, Brazil

Figure S1. Copaíba (*Copaifera reticulata* Ducke). (A) Tree individual in its naturais environment, highlighting the typical tall stature of the species. (B) Trunk perforation for oleoresin extraction, demonstrating the traditional tapping method. (C) Branch with compound leaves used for botanical identification. (D) Freshly collected copaíba oleoresin, showing its color and physical appearance.

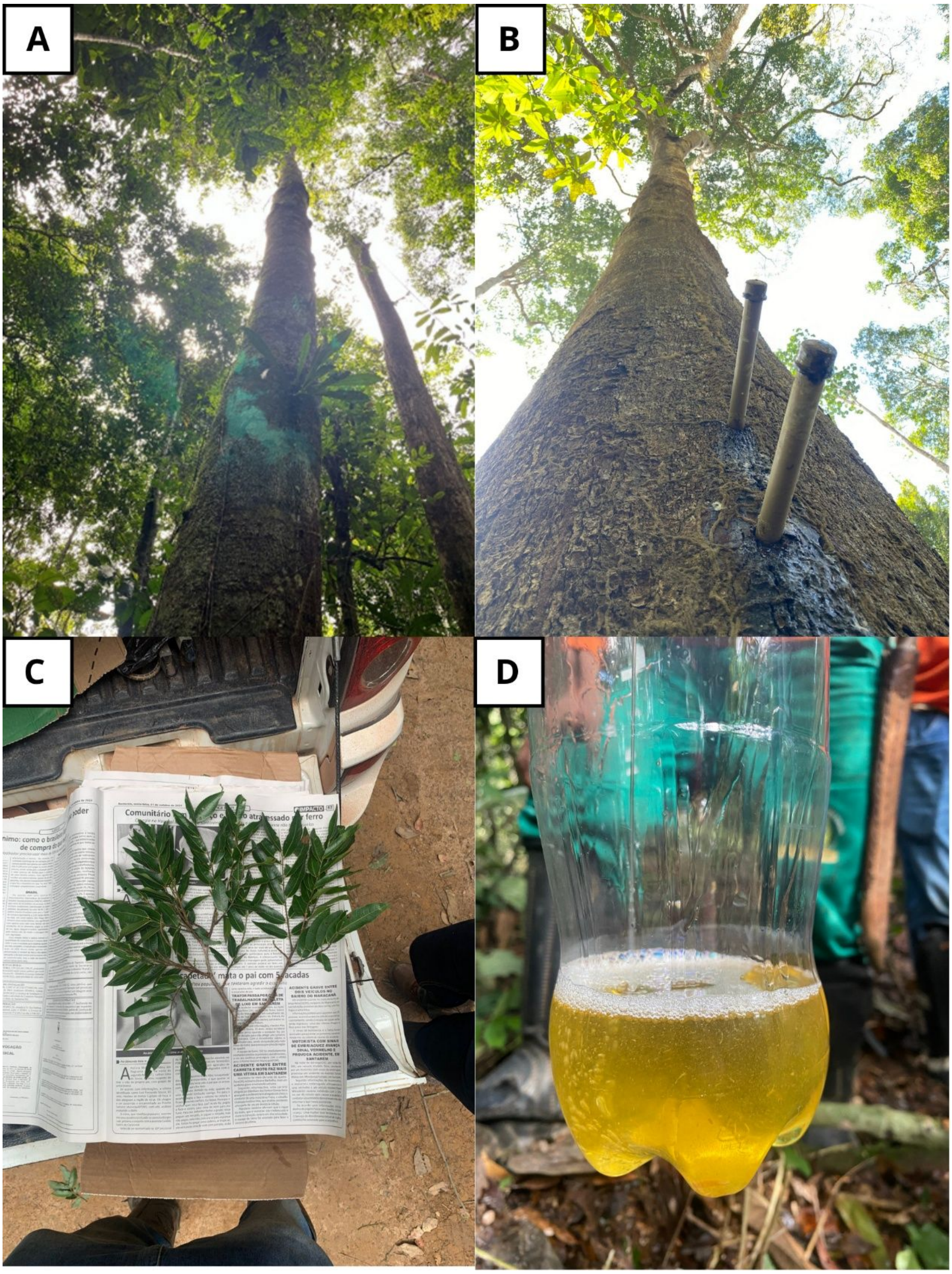

Figure S2. Processing and evaluation of copaíba oleoresin (*Copaifera reticulata* Ducke). (A) Hydrodistillation setup used to separate the resinous fraction from the volatile fraction. (B) Resinous fraction obtained after distillation, still associated with distilled water, showing phase separation. (C) Preparation and dilution of samples for experimental assays. (D) Larval bioassays performed to evaluate the biological activity of the samples.

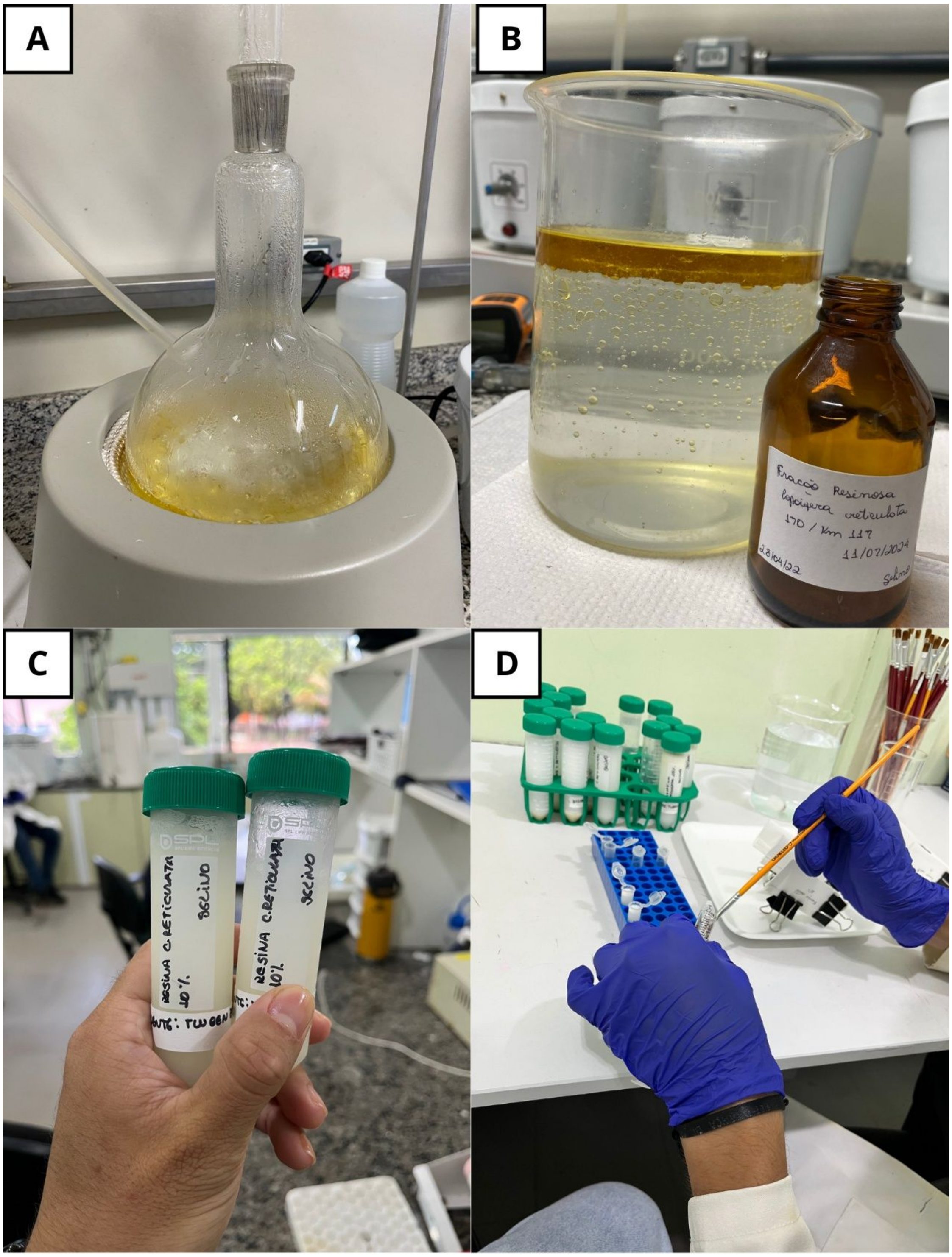

Figure S3. Acaricidal bioassay with copaíba oleoresin fractions (*Copaifera reticulata* Ducke). (A) Filter papers containing larvae after immersion in treatments, being transferred to a BOD incubator. (B) Experimental setup inside the incubator under controlled conditions. (C) Treatment with the resinous fraction, showing dead and desiccated *Rhipicephalus microplus* larvae. (D) Control treatment with Tween (diluent), where larvae remain alive and active.

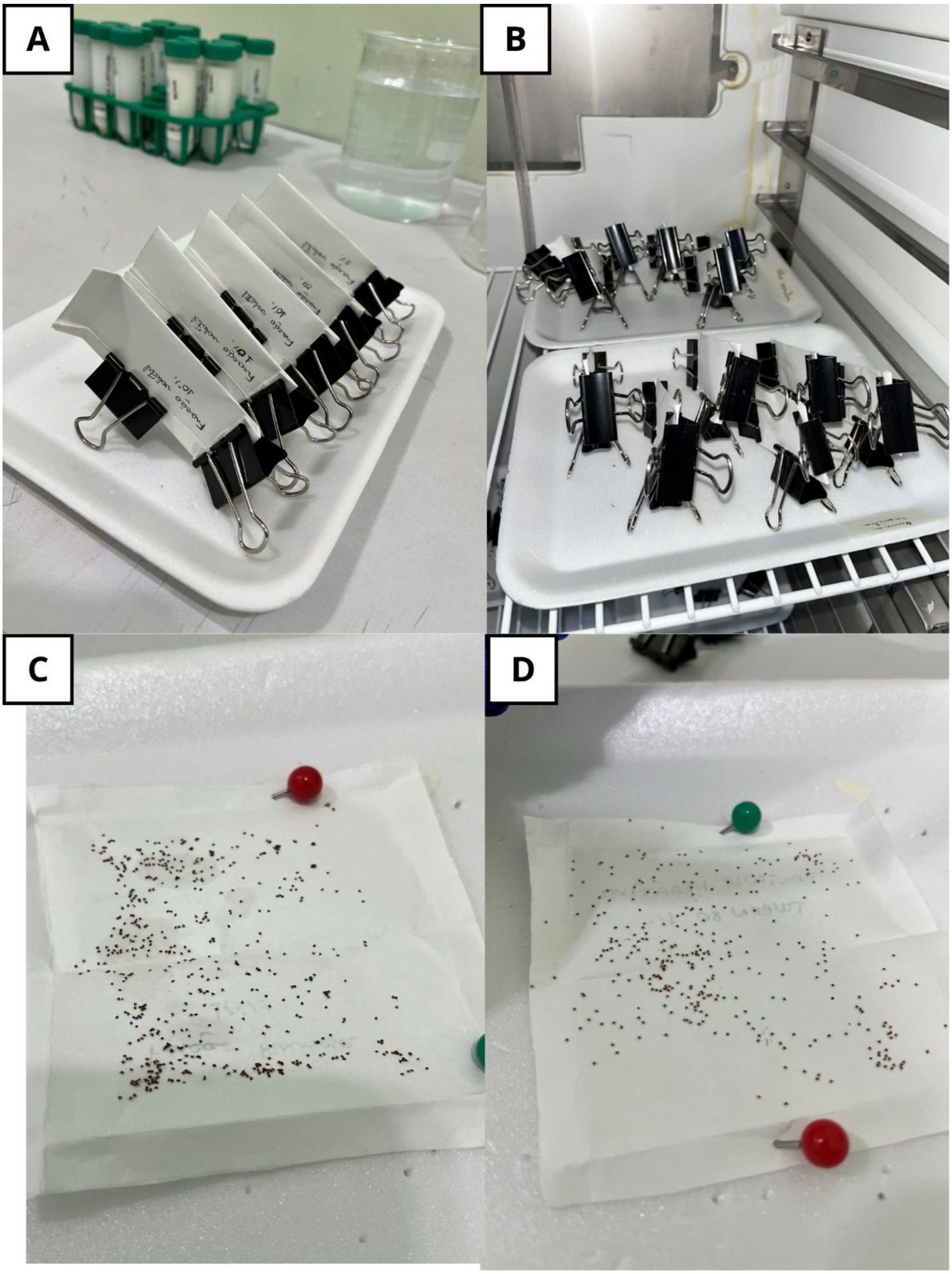

Supplement: Supplementary file 1 [file ao6c00834_si_001.pdf]
